# Hedgehog and Resident Vascular Stem Cell Fate

**DOI:** 10.1155/2015/468428

**Published:** 2015-05-06

**Authors:** Ciaran J. Mooney, Roya Hakimjavadi, Emma Fitzpatrick, Eimear Kennedy, Dermot Walls, David Morrow, Eileen M. Redmond, Paul A. Cahill

**Affiliations:** ^1^Vascular Biology and Therapeutics Laboratory, School of Biotechnology, Faculty of Science and Health, Dublin City University, Dublin 9, Ireland; ^2^School of Biotechnology, Faculty of Science and Health, Dublin City University, Dublin 9, Ireland; ^3^Department of Surgery, University of Rochester Medical Center, Rochester, NY 14642, USA

## Abstract

The Hedgehog pathway is a pivotal morphogenic driver during embryonic development and a key regulator of adult stem cell self-renewal. The discovery of resident multipotent vascular stem cells and adventitial progenitors within the vessel wall has transformed our understanding of the origin of medial and neointimal vascular smooth muscle cells (SMCs) during vessel repair in response to injury, lesion formation, and overall disease progression. This review highlights the importance of components of the Hh and Notch signalling pathways within the medial and adventitial regions of adult vessels, their recapitulation following vascular injury and disease progression, and their putative role in the maintenance and differentiation of resident vascular stem cells to vascular lineages from discrete niches within the vessel wall.

## 1. Introduction

Hedgehog signalling is a critical component of the transcriptional machinery that drives early vascular development [1], ischemia-induced postnatal neovascularization [2, 3], and the formation of larger and more muscularized blood vessels [4, 5]. Hedgehog components are also natively present within the medial adventitial boundary of normal adult vessels [6] and are recapitulated within the medial layer following vascular injury and repair [7, 8] raising the possibility that Hh signalling components may in part contribute to vascular disease aetiology and/or progression.

The Notch pathway embodies a cell-cell signaling system that primarily governs cell-fate choices, and more in particular controls the balance between commitment to differentiate and repression of differentiation while permitting, or even promoting, proliferation of the undifferentiated cells (reviewed [9]). Notch activation in neural crest-derived cells is required for vSMC development [10], and endothelial cell (EC) expression of the ligand Jagged-1 provides the Notch-activation signal [11]. Notch is also critical to defining vascular smooth muscle (SMC) phenotype [12, 13]. Notch components are recapitulated within the medial layer of vessels following vascular injury and repair [7, 14] raising the possibility that Notch components are also involved in vascular disease aetiology and/or progression.

Resident stem cell niches are generally regulated in specialized microenvironments by complex interacting signalling networks that provide cues for self-renewal, differentiation, migration, and retention. It has been known for some time that the Hh pathway is a crucial component for maintenance of stem cell niches where it is associated with the promotion of stem cell self-renewal [15, 16].

Vascular SMC biology has been at the forefront of cardiovascular disease research for the last 50 years. The classic concept of dedifferentiation and “phenotypic modulation” of vSMCs was first developed to explain vSMC proliferation and growth* in vitro* and phenotypic changes during vascular remodelling* in vivo* [17, 18]. However, recent studies have challenged this concept of “phenotypic modulation of medial vSMC” and have proposed an additional putative role for resident multipotent vascular stem cell- (MVSC-) derived SMCs from within vasculogenic zones of the vessel wall in the initiation and/or progression of vascular lesions [19].

In this paper, we review the current status of Hh signalling and its interaction with Notch and propose how recapitulation of Hh and Notch signalling components within the vasculature may act to coordinate SMC accumulation through the regulation of resident vascular stem self-renewal and differentiation to SMC and contribute to vascular disease by promoting vascular remodelling.

## 2. Hedgehog Biosynthesis, Secretion, and Signalling

**Figure 1:**
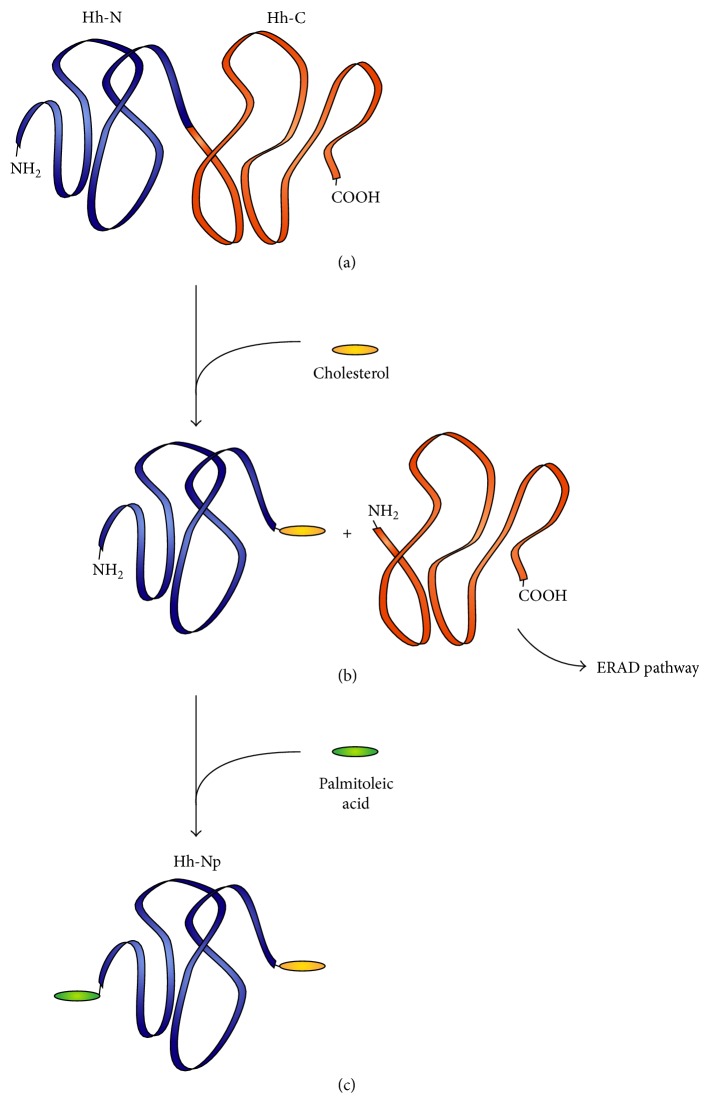
Posttranslational processing of Hh proteins. (a) The unprocessed Hh protein contains a 20 KDa N-terminal fragment (Hh-N) and a 25 KDa C-terminal self-splicing fragment (Hh-C). (b) Autoproteolysis occurs and a cholesterol molecule becomes covalently bound to the C-terminal of Hh-N. Hh-C is later degraded in the ER. (c) Addition of Palmitoleic Acid results in the fully processed form of the protein (Hh-Np) and mediates its transport to the cell membrane.

Since its discovery by Nüsslein-Volhard and Wieschaus in 1980, Hedgehog signalling (Hh) and its role as a key mediator of numerous cellular and developmental processes have greatly enhanced our collective understanding of numerous developmental pathways [20]. The Hh protein family consists of morphogenic molecules that are crucial in embryogenesis, postnatal morphogenesis, and general tissue homeostasis, acting in a dose-dependent manner or as inducing factors to control cell fate, proliferation, patterning, and cell survival (reviewed [21, 22]). Three Hh ligand proteins have been described in vertebrates, Sonic (Shh), Indian (Ihh), and Desert hedgehog (Dhh). While there is some redundancy, each almost exclusively mediates a particular developmental process such as vascular development [5], neural tube patterning [23], endochondral skeletal development [24], and spermatogenesis through the regulation of stem cell populations [25]. Briefly, the* Hh* gene family encode 45 KDa precursor proteins, which undergo autoproteolysis to produce a 20 KDa N-terminal fragment (Hh-N) that is covalently bound to a cholesterol molecule at its C-terminus and a 25 KDa C-terminal fragment (Hh-C) that is later degraded by the ER-associated degradation pathway (Figures 1(a) and 1(b)). Hh-N has been shown to be associated with all known Hh signalling activities while Hh-C is responsible for the self-splicing of the Hh protein. Hh-N undergoes further lipid modification by addition of a palmitoleic acid to the most N-terminal cysteine residue through an amide linkage to produce the fully processed form of the protein (Hh-Np), a modification that is catalysed by Skinny hedgehog acyltransferase (Figure 1(c)). These lipid modifications increase the hydrophobicity of the Hh protein and allow lipid tethering on the outer leaflet of the cell membrane.

Hh ligand secretion is accomplished via two distinct and synergistic cholesterol-dependent binding events, mediated by two proteins that are essential for vertebrate Hh signaling: the membrane protein Dispatched (Disp) and a member of the Scube family of secreted proteins. Cholesterol modification is sufficient for a heterologous protein to interact with Scube and to be secreted in a Scube-dependent manner. Disp and Scube recognize different structural aspects of cholesterol similarly to how Niemann-Pick disease proteins 1 and 2 interact with cholesterol, suggesting a hand-off mechanism for transferring Hh from Disp to Scube. Thus, Disp and Scube cooperate to dramatically enhance the secretion and solubility of the cholesterol-modified Hh ligand (reviewed [21, 22]).

The Hedgehog (Hh) signalling pathway relies on the primary cilium to regulate tissue patterning and homeostasis where ciliary localization and trafficking of Hh components lead to pathway activation and regulation. Recent studies reveal specific roles of discrete ciliary regulators, components, and structures in controlling the movement and signaling of Hh components. Active Hh signalling is associated with increased levels of Hh components along the primary cilium or in a ciliary subdomain [23, 26]. On ligand binding to its membrane receptor Ptch1, a dramatic reduction in Ptch1 expression ensues concomitant with an increase of ciliary Smo levels [27, 28]. Hence release of Hh-bound Ptch1 from the cilium allows for Smo entry through dynamic movements of Ptch1 and Smo that occur even in the absence of active Hh signaling.

**Figure 2:**
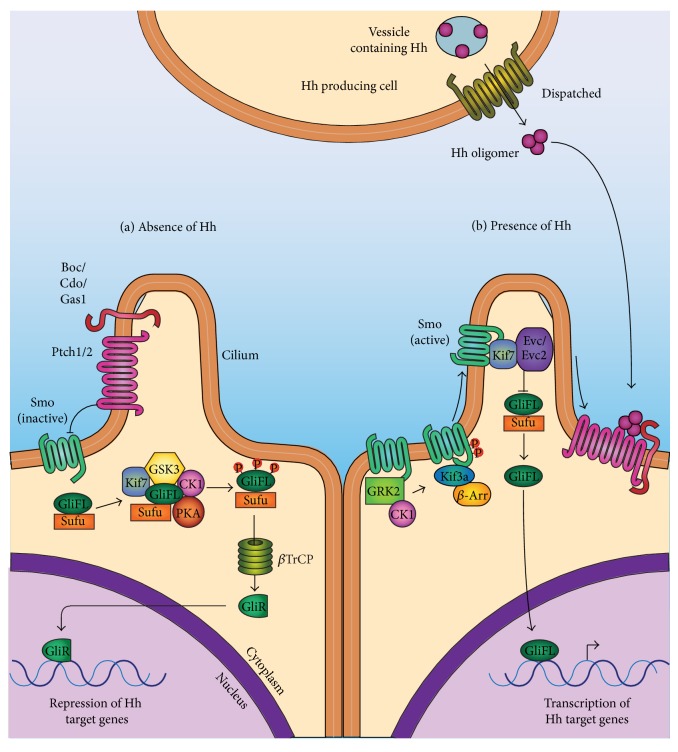
Hh signalling in vertebrates. (a) In the absence of Hh, the Hh receptor, Ptch, represses Smo. Sufu binds to the Gli-FL transcription factors, sequestering them in the cytoplasm. Sufu and Kif7 then mediate the phosphorylation of Gli-FL by recruiting PKA, GSK3*β*, and CK1*α*. This signals for the proteolytic cleavage of Gli-FL by *β*TrCP, resulting in the degradation of the C-terminal and formation of Gli-R. Gli-R then localizes to the nucleus and represses the expression of Hh target genes. (b) In the presence of Hh, Hh binds to Ptch, antagonizing its repressor function. Smo becomes phosphorylated by CK1*α* and GRK2 and promotes *β*-arrestin and Kif3a-dependent trafficking of Smo to the cilium. With the aid of Kif7, Smo then promotes the disassembly of the Sufu-Gli-FL complex. Gli-FL then localizes to the nucleus to activate target gene expression and is later degraded by Spop.

Downstream signalling of the hedgehog pathway is stimulated when Hh binds to the integral-membrane protein Patched (Ptch). There are two distinct homologs of the Ptch gene (Ptch1, Ptch2) in vertebrates that are differentially expressed during development.* Ptch1* ^−/−^ is embryonically lethal in mice. In the absence of Hh, Ptch catalytically represses the seven-transmembrane protein Smoothened (Smo) [29]. The exact mechanism of how Ptch represses Smo is incompletely understood; however, it may involve oxysterols [30]. The subsequent binding of Hh to Ptch antagonizes its repressor function. In vertebrates, the transcription of Hh target genes is controlled by Gli1–3 transcription factors [31]. Gli1 and Gli2 primarily act as transcriptional activators while Gli3 is the principal transcriptional repressor. However, Gli2 and Gli3 have been shown to possess both activator and repressor functions [32]. In the absence of Hh, the full-length Gli (Gli-FL) transcription factors are cleaved to their repressor forms through phosphorylation cascades that cause retention of Gli-FL proteins in the cytosol. This leads to the ubiquitylation, cleavage, and degradation of the C-terminal peptides, generating the repressor forms of the proteins (Gli-R). Gli-R proteins then localize to the nucleus and repress target gene expression [33] (Figure 2(a)). Upon Hh binding of the Ptch receptor, hyperphosphorylation and activation of Smo ensues before the disassembly of the microtubule-binding Sufu-Gli-FL complex occurs. Smo mediates the disassembly of the Sufu-Gli-FL complex, thus allowing the Gli-FL proteins to localize to the nucleus and activate transcription (Figure 2(b)). Histone deacetylase 6 (HDAC6) appears essential for both full Hh pathway activation and complete repression of basal Hh target gene expression through its impact on Gli2 mRNA and GLI3 protein expression [34].

A number of other signal pathways are also regulated by the Hh-ligands. These include those involving Rac1, Protein kinase C, and MAP kinase, which respond to Hh signalling in a Smo-dependent manner [35–37]. Some of these appear to modulate the output of the Hh- pathway itself by modifying the activity of specific components of the canonical pathway. Importantly, Ptch1 may also mediate Hh signalling independently of Smo or other components of the canonical pathway [36, 38].

## 3. Hedgehog Signalling and Cardiovascular Development

Analysis of Shh and Ihh expression during different time-windows of mouse embryogenesis has demonstrated the importance of Hh signalling in the development of the mouse vasculature (reviewed [39]). Hh target genes include angiogenic growth factors such as vascular endothelial growth factor (VEGF) and angiopoietins. These factors are activated in a Hh-mediated signalling hierarchy to regulate primitive and definitive hematopoiesis, the late stages of vasculogenesis, angiogenesis, and arterial-venous identity [2, 5, 39, 40].

**Figure 3:**
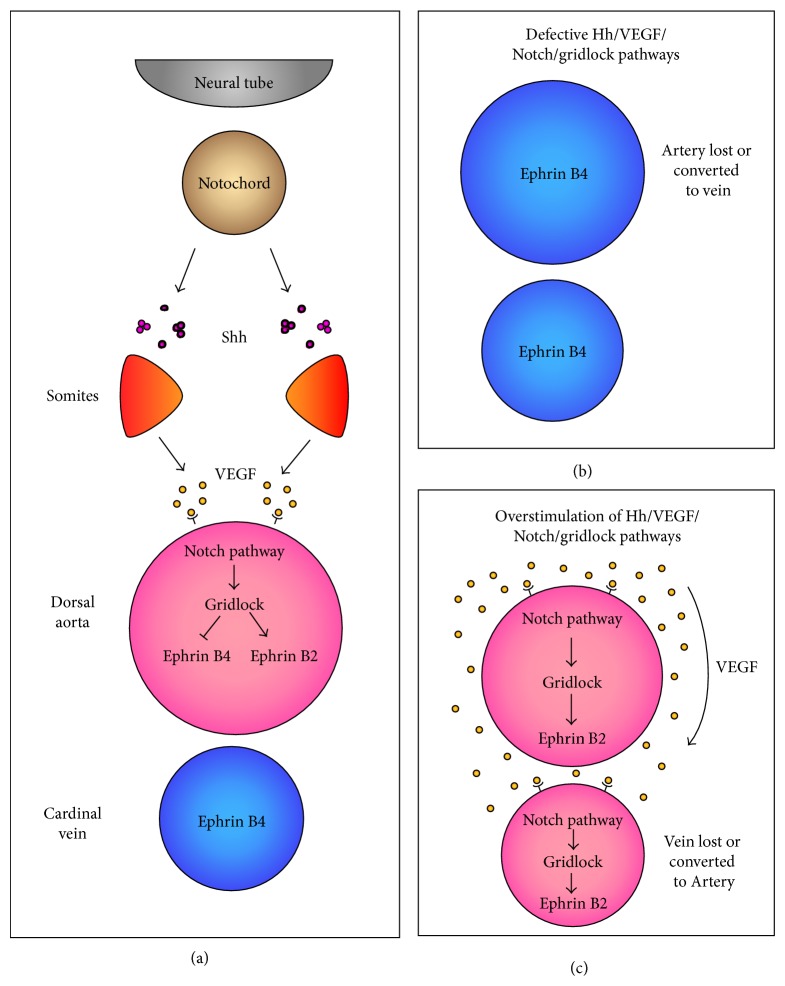
Signalling cascade involved in determining arterial-venous identity in zebrafish. (a) Shh from the notochord induces* vegf* expression in somites, promoting arterial identity of endothelial cells in the aorta. The arterial ECs then stimulate Notch signalling in the surrounding cells resulting in an upregulation of gridlock and thus ephrin B2, conferring arterial identity of the aorta. (b) Impairment of the signalling hierarchy responsible for arterial-venous identity results in either loss of arterial identity in vessels or loss of arterial vessels entirely. (c) Overstimulation of one or more of the pathways in the signalling hierarchy responsible for arterial-venous identity results in either gain of arterial identity in venous vessels or loss of venous vessels entirely.

Arterial-venous identity is controlled by Hh signalling whereby inhibition of Hh signalling with cyclopamine in zebrafish yolk sacs results in the formation of a single longitudinal axial vessel that shows markers of venous identity [41] (Figure 3). Functional studies using* sonic you (Syu)*, the gene that encodes the zebrafish homolog of Shh, have determined that Hh signalling mediates the arterial-venous determination by regulating the Notch pathway via VEGF (Figure 3). Genes involved in the Notch pathway, such as* Notch1*,* Notch4*,* Jag1*,* Jag2*, and* Dll4*, are expressed in arterial but not venous vessels [42]. During development, the notochord and floor plate act as Shh sources to promote vessel formation through upregulation of VEGF, Notch, and ephrin B2 pathways. Notch signalling leads to upregulation of* ephrin B2* and* Gridlock*, molecules required for arterial identity, and downregulates* ephrin B4*, a molecule that is expressed by cells of venous identity [41].

**Figure 4:**
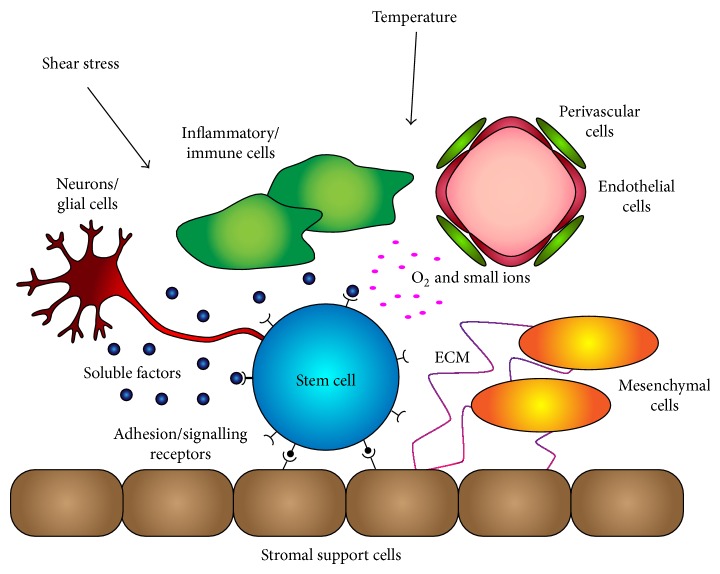
Stem cell niche. The stem cell niche is composed of multiple niche support cells, such as stromal and mesenchymal cells, that provide stimulus to a small subpopulation of stem cells. The stem cells are responsive to cellular and acellular regulatory components within the niche. These regulatory components control the fate of the stem cells, be it proliferation, self-renewal, differentiation, fate, programmed cell death, retention, or migration.

Hh signalling is significantly involved in the maintenance of stem cells during development and throughout adult life. It is particularly important in the regulation of resident stem cells within tissues that have high cellular turnover, such as Shh/Ihh in the intestine [43] and Shh in the hematopoietic system [44] and in epidermal tissue homeostasis [45]. In most tissues, stem cells are maintained in a specialized microenvironment, termed the stem cell niche (reviewed [46]) (Figure 4). The surrounding niche support cells are crucial in maintaining stem cell niche homeostasis by providing structural support, producing cell signalling molecules, and regulating adhesive interactions. The Hh pathway along with the Wnt, Notch, and Bone morphogenetic protein (BMP) signalling pathways make up the signalling networks involved in stem cell niche homeostasis [47, 48]. Physical parameters such as temperature, pressure, hypoxia, and shear stress are also involved in the maintenance of stem cell niche homeostasis [49–51]. While the specific environmental cues differ for each particular stem cell niche, the Hh signalling network mediates stem cell proliferation, self-renewal, differentiation, fate, and programmed cell death, survival, retention, and migration (reviewed [52]) (Figure 4).

## 4. Hh Signalling and the Vasculature

In addition to its provascular forming properties during embryogenesis, an Hh-mediated angiogenic signalling hierarchy is reactivated during postnatal vascular repair processes in pathological conditions such as ischemia, tissue regeneration, inflammation, and cancer [2, 3, 7, 8, 53].

Initial studies demonstrated that Shh and VEGF mediated angiogenesis in mouse ischemic limb models, a process attenuated in the presence of a Hh inhibitor [2]. Treatment with exogenous Shh in these ischemic models results in improved angiogenesis [2, 3]. Similar studies by Surace et al. reported retinal angiogenesis following upregulation of Shh and VEGF after ischemia in mice, angiogenesis that was attenuated by treatment with the Hh inhibitor cyclopamine [54]. Similar proangiogenic effects have been reported with other Hh ligands [40]. As mural cells (MC) such as pericytes and/or SMCs are recruited to endothelial sprouts during angiogenesis and their proliferation and migration towards ECs are dependent on a precisely orchestrated gradient of soluble chemotactic factors generated from ECs in their microenvironment, it is noteworthy that Shh upregulates MC-related markers during the formation of microvessel-like structures* in vitro* [55] suggesting a putative role for Shh in vessel maturation. Indeed, at the cellular level, recombinant Shh induces SMC and pericyte proliferation and survival [7, 53, 56, 57] and increases the endogenous effect of PDGF-BB in promoting vessel maturation by enhancing SMC and pericyte migration towards PDGF-BB-producing ECs [58]. Shh may also stimulate bone marrow-derived BM-EPC proliferation and migration and VEGF production via a PI3-kinase/Akt signalling pathway to promote neovascularization to ischemic tissues [59].

The majority of Hh signalling components are confined to the adventitial layer of normal adult vessels where both Shh and Ptch1 receptors are preferentially located [6, 7]. An emerging concept is that the vascular adventitia acts as a focal point for the retrieval, integration, storage, and release of key regulators of vessel wall function. In response to stress or injury, resident adventitial cells can be activated and reprogrammed to exhibit different functional and structural behaviours. Several investigators have shown that the adventitial compartment may be considered the principal injury-sensing tissue of the vessel wall. In response to vascular stresses such as overdistension and hypoxia, the adventitial fibroblast is activated and undergoes phenotypic changes, which include proliferation, differentiation, upregulation of contractile and extracellular matrix proteins, and release of factors that directly affect medial smooth muscle cell tone and growth and that stimulate recruitment of inflammatory and progenitor cells to the vessel wall [60–62] and that have been reviewed elsewhere [63]. The molecular factors that dictate these responses include Wnt, BMP, and Notch signaling, all of which have been implicated in controlling cell-cell interactions within other niche environments and are critical for maintaining progenitor cells capable of contributing to tissue homeostasis and repair [64–66].

Importantly, the colocalisation of Ptch1^+^/Shh^+^ and Gli_2_^+^ cells with resident vascular stem cells within the adventitial layer of arterial vessels [7, 8] suggests that Shh signaling within the adventitia may regulate adventitial progenitor cell proliferation, self-renewal, and survival, at least in adventitia surrounding the aortic root and thoracic aorta [6]. It is also highly likely that other soluble factors in addition to Shh play an important role in dictating the adventitial stem cell behaviour.

## 5. Resident Vascular Stem Cell Niches

Murry et al. originally provided the first evidence for a monoclonal origin of SMCs in atherosclerotic lesions [72]. This seminal finding was later corroborated by the identification of ATP-binding cassette transporter subfamily G member 2- (ABCG-2-) expressing stem cells in the medial layer of the mouse aorta and femoral artery, suggesting a particular involvement of a developmental subpopulation of medial SMC in the aetiology of vascular lesions (Table 1) [73]. The discovery of hematopoietic stem cells in blood initially raised the possibility of a bone marrow-derived stem cell origin of neointimal SMCs following injury or disease progression [74]. However, although a number of studies have since implicated bone marrow- (BM-) derived progenitor cells in neointima formation (reviewed [75]), these results remain controversial since BM-derived stem cells cannot differentiate into mature SMCs in neointimal lesions when tracking them using a transgenic model with MYH11 as a reporter [76]. In contrast, the evidence for a role of resident vascular stem cells in vessel remodelling is more robust [19, 63, 68, 69, 73, 77].

**Figure 5:**
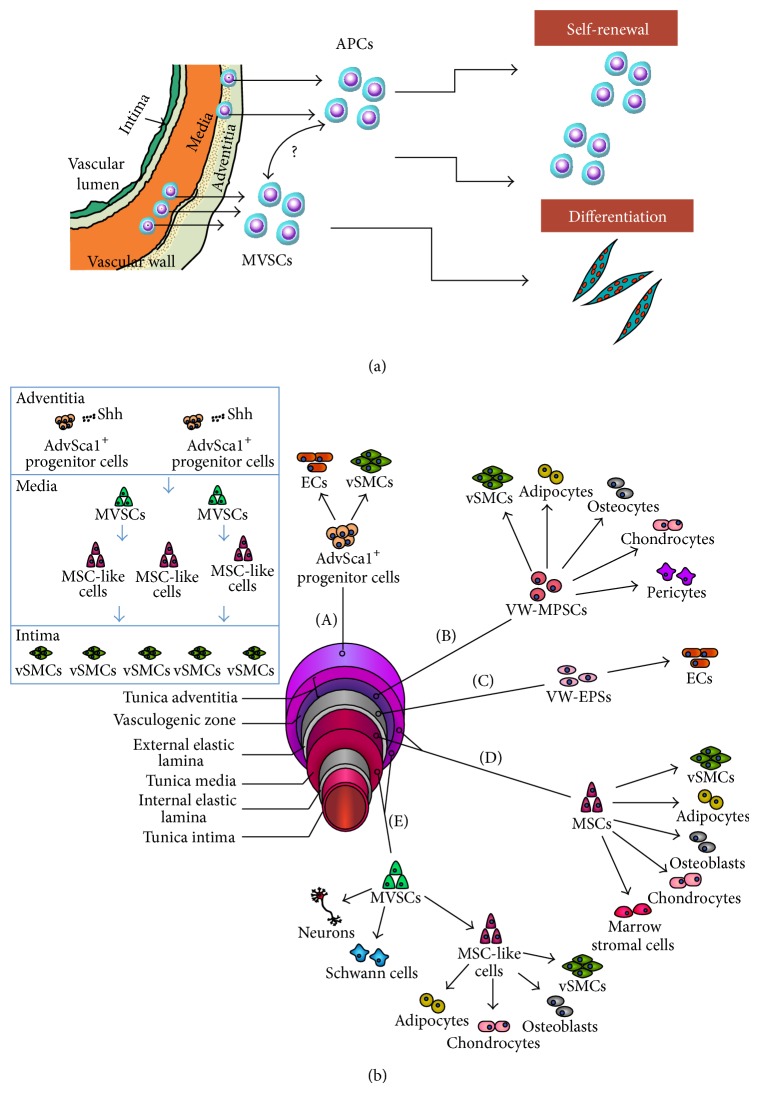
(a) APC and MVSC locations within the blood vessel wall. AdvSca1^+^ progenitor cells reside in the tunica adventitia and MVSC reside within the medial adventitial boundary and are both capable of transition to SMCs. (b) Differential potential of the resident stem and progenitor cells present in the blood vessel. AdvSca1^+^ progenitor cells (APC) reside in the tunica adventitia and have the ability to differentiate into ECs and vSMCs. VW-EPS were isolated from the vasculogenic zone of the tunica adventitia. These cells can differentiate into vSMCs, adipocytes, osteocytes, chondrocytes, and pericytes. VW-EPS were found in the external elastic lamina and were shown to form capillaries in culture and increase expression of mature EC markers during the process. MSCs have been isolated from both the tunica media and the tunica adventitia and have been shown to differentiate into adipocytes, osteoblasts, chondrocytes, marrow stromal cells, and SMCs. MVSCs were present in the tunica media and have the ability to differentiate into neurons, Schwann cells, and MSC-like progenitor cells. Once the MVSCs have transitioned into a MSC-like state they are capable of differentiating into adipocytes, osteoblasts, chondrocytes, and vSMCs. Inset: Hedgehog regulation of progenitor cells present in the vessel wall.

The maintenance and control of the integrity of the vascular wall is required to provide an appropriate response to injury and ageing. Over the last 10 years, stem cell niches within vasculogenic zones of the vessel wall have emerged as potential sources of regenerative SMCs. Indeed, resident “calcifying vascular cells” (CVCs) had been implicated in atherosclerotic lesions and neointimal formation for some time [68]. Originally, CVCs were first demonstrated to differentiate into osteoblasts, resulting in arterial calcification; however, later analysis demonstrated that they were in fact multipotent [68]. These cells were positive for CD29, CD34, and CD44, could self-renew, and shared an antigen presentation profile similar to mesenchymal stem cells (MSCs). A number of studies have since reported the presence of medial MSCs and MSC-like cells within human blood vessels, some of which share similar surface antigen profiles, such as CD13^+^, CD29^+^, CD44^+^, CD90^+^, CD34^−^, and CD31^−^, and similar differential capabilities [69, 71, 77] (Figure 5(b)).

Other multipotent stem cells that reside within vasculogenic zones of the adventitial and medial layers of the vessel wall have also been reported [73, 78, 79] (Figure 5(a)). Klein et al. described the presence of vascular wall-resident multipotent stem cells (VW-MPSCs) within the adventitial vasculogenic zone of human arteries that were positive for a number of MSC surface markers, such as CD44, CD105, CD73, CD90, CD29, and Stro1, and the expression of Oct4 and Sox2, two transcription factors associated with stem cell activity. They were multipotent and, furthermore, when cocultured with HUVEC cells in matrigel, they covered the capillary structures that were formed by the HUVEC cells.* In vivo* studies confirmed their ability to contribute to vessel morphogenesis in mice, a process that was enhanced by the application of VEGF, FGF-2, and TGF-*β*1 [79]. More recently, a role for certain HOX genes in regulating their differentiation into SMCs through epigenetic mechanisms has been reported and may be critical for understanding how VW-MPSC–dependent vascular disease processes such as neointima formation and tumour vascularization prevail [80].

Another progenitor cell population within the adventitial medial boundary of the vessel wall was also described in rodents [67]. These adventitial progenitor stem cells (APCs) are positive for stem cell antigen-1 (Sca1) in mice, stage-specific embryonic antigen-1, c-Kit, and Flk1 [67] and were not of bone marrow (BM) origin but have the ability to differentiate into SMCs in the presence of PDGF and ECs in the presence of VEGF* in vitro*, demonstrating their multipotency (Figure 5(b)) Table 2 [70]. They are also capable of differentiating into neointimal vSMCs* in vivo* [67]. Passman et al. later determined, using Wnt1-Cre/LoxP-LacZ mice, that these cells from the common carotid arteries, ductus arteriosus, pulmonary trunk, and proximal aorta do not arise from the neural crest [6]. Importantly, however, although a human ortholog of Sca1 has yet to be identified, the in-depth study of the mechanisms that underlie Sca1 function in mice has been critical to further our understanding of Ly6 family proteins, glycosyl phosphatidylinositol-anchored cell surface protein (GPI-Aps), and, most importantly, human stem cell biology [87].

The most recent studies have described a resident medial stem cell population and raised the intriguing possibility that differentiation of these multipotent vascular stem cells (MVSCs) may contribute in part to vascular remodelling and disease progression [19]. These cells do not appear to be the same as the “side population” previously isolated from tunica media of adult mice aortas using flow cytometry [73]. Surface marker analysis found that MVSCs are derived from the neural crest and so express astrocyte marker, S100*β*, the neural stem cell marker, Sox10, and the endoderm marker, Sox17 [19]. Additionally, MVSCs did not express Sca1 or CD146 under specific conditions, suggesting a distinction from the previously described AdvSca1^+^ progenitors cells and medial MSC-like stem cells present in the vessel wall. Once maintained in culture, they are multipotent and cloneable, have telomerase activity, and can differentiate into chondrocytes, adipocytes, osteoblasts, neural cells, and SMCs* in vitro* (Figure 5(a)).

The majority of medial differentiated SMCs within the uninjured vessel wall are positive for MYH11 [17, 19, 88, 89]. However, following vascular injury or stenting, MYH11 levels are significantly reduced and the cells adopt a more embryonic-like phenotype [90, 91]. Lineage tracing studies have shown that both resident MYH11^−^ negative medial MVSC-derived SMCs [19] in addition to dedifferentiated SMCs can populate the tunica media after injury and contribute to vascular remodeling [92, 93]. Corroborating studies defining differentiated SMCs as the source of neointimal SMCs using epigenetic histone modifications of the MYH11 gene locus in SMCs* in vitro* and* in vivo* have also been presented [89]. Moreover, despite differences with the methodology of the lineage tracing experiments (constitutive [19] versus inducible [92, 93] systems) where it is shown whether (neo)intimal cells are derived from differentiated SMC or not, it is clear that these cells in animal models [19] and humans [94] are multipotent. Furthermore, even when the majority of MYH11 positive cells are tracked in* Cre-LoxP* transgenic mice to the neointimal layer using confocal microscopy, as many as 60% of cell population in some vascular sections are MYH11^−^ negative following vascular injury [93]. The fact that MYH11^−^ negative MVSC-derived SMCs are present in the vessel wall raises the further possibility that most SMCs in culture are also derived from a resident medial stem cell population during the culture process underscoring the potential importance of this medial MVSC population to vascular biology in general [95–97].

Consistent with the niche function of both adventitial and medial vasculogenic zones, APCs and medial MVSCs have been reported in rodents [19, 63, 67] and human vessels [19, 79, 80, 98], respectively. Moreover, these cells exhibit MSC-like behaviour [19, 79] as they transition down a vascular lineage. More importantly, these resident vascular stem cells respond to Hedgehog [6] and Notch [19] activation, respectively, by transitioning to a vascular lineage [19, 99] supporting a putative role for a Hh-Notch axis in controlling stem cell renewal and/or transition from these vasculogenic zones. The resolution of the ultimate origin of cultured SMCs* in vitro* and neointimal SMCs within vascular lesions* in vivo* awaits a more rigorous use of a conditionally regulated SMC lineage tracing systems using the MYH11 gene in addition to other specific genes for differentiated SMC, such as SM22*α* and smoothelin B [89]. In addition, conditionally regulated stem cell lineage tracking systems using specific traceable markers for both adventitial progenitors and MVSCs in combination with high-resolution confocal analyses will greatly assist in deciphering the subsequent fate of APCs and MVSCs at multiple time points after vascular injury or induction of disease. In this context, recent studies have confirmed that Nestin^+^ and Sca1^+^ cells are significantly increased following vascular injury [100]. While the authors attribute this to TGF*β* as an injury-activated messenger essential for the mobilization and recruitment of MSCs to participate in tissue repair/remodelling, a role for resident Nestin^+^ and Sca1^+^ cells cannot be ruled out.

Finally, there is also increasing evidence that supports the plasticity of differentiated SMCs, and it is highly likely that SMCs may also dedifferentiate or transdifferentiate in response to specific environmental cues to a more plastic stem-like state. Such plasticity of dedifferentiated SMCs may be the ultimate driver for various vascular pathologies. Indeed, murine SMCs are capable of transdifferentiation to macrophage-like cells following cholesterol treatment [101], in addition to osteogenic and skeletal lineages* in vitro* [102–104]. Moreover, human SMC are capable of transdifferentiation to a neural stem-like phenotype typical of MVSCs if treated with a combination of sex steroids [105]. Finally, TGF-*β*/Smad3 stimulates stem cell/developmental gene expression and SMC dedifferentiation* in vitro* [106] further underscoring the plasticity of SMCs, at least in culture.

It is therefore likely that a delicate balance exists between stem cell differentiation and self-renewal to drive vessel regeneration, resulting in ordered layers of functional differentiated SMCs and residual stem cells responsible for renewal and repair. Such a scenario has been documented for prostate cancer (reviewed [107]). In addition, mechanical micro environmental cues and stimuli that are known to be correlated with vascular disease progression also have an impact on stem cell differentiation and renewal through similar signalling pathways involving Notch, TGF-*β*1, and Hh [53, 108, 109].

## 6. Hedgehog: Notch Signalling Axis and Vascular Remodelling

The clear importance of Hedgehog (Hh)/Notch signalling axis in vascular development [10] suggests that similar signalling patterns using VEGF-A, angiopoietins (Ang-1,2), and Notch may prevail in adult cells during regeneration and repair following injury or disease [7, 8, 14, 110]. Mice genetically deficient in Hh proteins have a smaller and less-organized capillary plexus, owing to their inability to undergo vascular remodelling during development [111]. Indeed, given that Hh signalling is involved extensively in arterial/venous identity via a Hh/VEGF/Notch dependent and independent axis [10, 41], it is not surprising that elements of this important axis are recapitulated in adult vascular cells after injury [7, 8, 14, 53].

Several groups have demonstrated that Hh signalling components promote changes in SMCs phenotype and growth* in vitro* [8, 53, 56, 57], yet the predominant target cell for Shh in vascular tissue* in vivo* in uninjured vessels is the adventitia [6]. The fact that Shh responsive cells are primarily confined to the adventitial boundary and colocalise with Sca1^+^ progenitor stem cells in normal vessels only to reappear within the media and intima following vascular injury [7, 100] is instructive. It may underlie a putative role for Hh in controlling stem cell renewal of the Sca1^+^ population at the adventitial boundary and/or transition of resident stem cells within the medial layers of the blood vessel wall. Indeed, Sca1^+^ cells in late embryonic and early postnatal mice require Shh signalling for their self-renewal since a restricted domain of Shh signalling has been localized to the arterial adventitial APC niche within the artery wall [6]. These APC progenitor stem cells were reported not to be derived from either bone marrow or neural crest (different to MVSCs), and their origin is currently unclear [6]. This Shh requirement for APCs is interesting, because Shh signalling maintains stem and progenitor cell niches in other developmental contexts, in particular within the brain [112]. Sca1^+^ progenitors also notably contribute to neointima formation in vein grafts from atherosclerosis-prone apolipoprotein E-knockout mice [67] and cells mobilized in response to vascular injury where they acquire a high proliferative index when activated following injury [100, 113]. Moreover, these studies in mice are consistent with adventitia-localized progenitor cells in human vessels [80]. Shh is also upregulated in autogenous vein grafts obtained from mice undergoing restenosis with extensive remodelling [56] in addition to chronic graft-versus-host disease (cGVHD). Treatment with LDE223, a highly selective small-molecule antagonist of the hedgehog coreceptor Smoothened (Smo), abrogated the activation of hedgehog signalling and protected against experimental cGVHD independent of leukocyte infiltration [114].

The functional importance of activation of Hh positive cells in the pathogenesis of vascular proliferative disease is further validated following specific knockdown of the Hh signalling pathway within the vessel wall following perivascular delivery of Ptch1 siRNA [8]. Although targeted local inhibition of Hh signalling in ligated carotid arteries* in vivo* attenuated medial intimal hyperplasia concomitant with a reduction in adventitial volume [8], it is not clear at present whether this inhibition is associated with a genuine reduction in the number of Sca1^+^ progenitor cells and/or differentiation of stem cells to SMC thereby decreasing the number of medial and intimal SMCs present within the lesion. Hh signalling is known to promote the growth of human pulmonary SMCs following hypoxic injury [57]. Importantly, overexpression of Shh or exogenous Shh induces autophagy of vSMCs through activation of AKT, while cyclopamine, another Hh inhibitor, also attenuated neointima formation in murine carotid arteries following ligation [115].

The different patterns of Notch receptor expression in vascular tissue suggest that Notch receptors may also have distinct roles in SMC function [116]. However, Notch1, rather than Notch3, mediates SMC accumulation and neointimal formation following vascular injury [14, 116]. In uninjured vessels, Notch1 expression is confined to the endothelial layer [7, 116] suggesting that few Notch1 responsive cells are present within adventitial and medial vasculogenic zones. Nevertheless, perivascular depletion of Notch1 receptors in injured vessels attenuates SMC accumulation and neointimal formation following vascular injury [14, 116]. The key question then arises as what cell populations are targeted by the various interventions to inhibit hedgehog and Notch signalling and ameliorate injury-induced vascular remodelling? In this context, it is known that Shh increases VEGF and stromal cell-derived factor 1 expression in fibroblasts while the medium from Shh-transfected fibroblasts increased the migration, proliferation, and tube formation of bone marrow-derived progenitor cells (BMPCs) [117]. Sca1^+^ also differentiates to SMC by adopting an elongated, mesenchymal cell shape and expressing SMA, SM22*α*, calponin1, and SM-MHC [6]. However, when these same cells were incubated with VEGF-A (10 ng/mL, 9 days), PECAM1-positive endothelial cell clusters were apparent whereas when incubated with BMP2 (50 ng/mL, 20 days), cells differentiated down an osteogenic lineage [6]. Intriguingly, treatment of these cells in primary explant culture with Shh or the Hh signalling inhibitor, cyclopamine, provided evidence that Hh signalling also mediates mitogenic and survival responses in these progenitor stem cells [6]. Moreover, cyclopamine appeared to inhibit the number of SMA positive cells suggesting that Hh controls Sca1^+^ transition to SMC* in vitro* [99]. Finally, inhibition of Hh signalling* in vivo* using a neutralising antibody (5E1) strategy resulted in decreased plasma cholesterol levels but increases atherosclerosis due to enhanced lipid uptake by macrophages [118].

**Figure 6:**
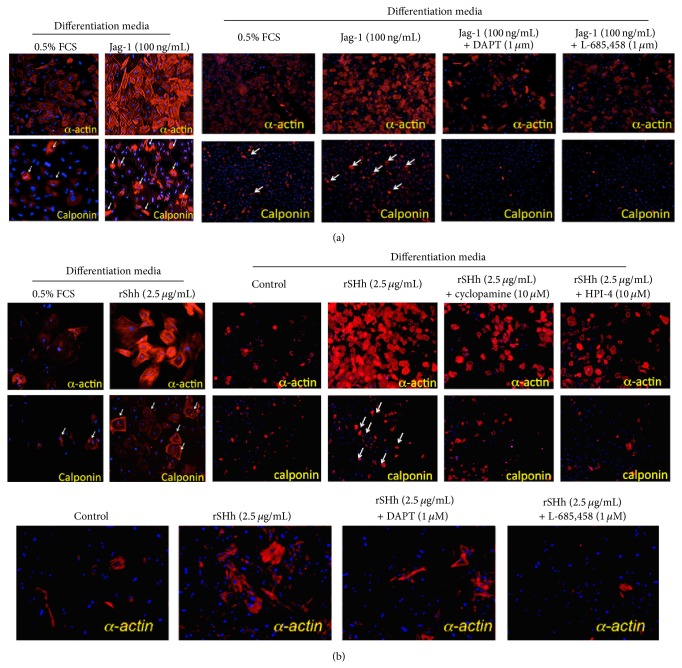
Hedgehog and Notch control of mesenchymal stem cell (MSC) transition to vSMC. Rat MSCs were treated with recombinant rSHh or the Notch ligand, Jagged-1, for 5 days in 0.5% FCS in the absence or presence of Hh (cyclopamine and HPI-4) and Notch inhibitors (*γ*-secretase inhibitors, DAPT and L-685,458). Vascular SMC differentiation was assessed by determining smooth muscle *α*-actin (SMA) and calponin-1 (CNN1) positive cells.

Transforming growth factor-beta 1- (TGF-*β1-*) induced expression of the Notch ligand Jagged 1 is known to act on MSCs and promote vSMC marker expression [108]. While Notch is prominently expressed in the side population Sca1^+^ cells from various tissues and plays a significant role in their self-renewal [119], Notch1 receptors are not present in the Sca1^+^ adventitial vasculogenic zone* in vivo* [7, 116]. In our hands, recombinant rShh and jagged 1 stimulation of a bone marrow-derived MSC population resulted in transition to SMC and is attenuated by cyclopamine and HPI-4, both Hh inhibitors, and by the *γ*-secretase Notch signalling inhibitors, DAPT and L-685,458, respectively, implicating a Hh-Notch role in MSC-like cell transition to SMC (Figure 6). Considering that both APCs and MVSCs exhibit MSC-like behaviour, these findings increase the distinct possibility that Hh signalling and its downstream targets, such as TGF-*β*1, VEGF, and Notch, control resident vascular stem cell progenitor and MSCs-like transition to SMCs. While local targeted inhibition of Hh and Notch pathways* in vivo* attenuates vascular remodelling in carotid artery injury models[8, 14], a direct link to adventitial Ptch1^+^ and/or Sca1^+^ positive cells and medial Sox10/Sox17/S100*β* ^+^ MVSCs through lineage cell fate mapping in mice following vascular injury is now required. In this context, Song Li's group at Berkeley, CA, who were first to report MVSCs [19] have preliminary data to suggest that Sox10^+^ cells in* Cre-LoxP* transgenic mice locate to the injured femoral artery following wire-induced injury (unpublished communication).

While a direct role for Notch in the regulation of medial MVSCs has not yet been fully investigated, a putative role is strongly implicated as activation of Notch signalling in MVSCs directs their differentiation into mature SMCs when cocultured with OP9-Delta1 Notch ligand presenting feeder cells [19]. This is not surprising as Notch has recently been shown to control differentiation of SMCs from local precursors during both embryonic and adult arteriogenesis [120]. The effect of Notch signalling on SMC of neural crest and somitic origin has been studied using mammalian and avian models, respectively [10, 11, 121]. A Tie1^+^ precursor appears to represent an immediate vascular precursor predisposed to a SMC fate in the presence of Notch activation, regardless of the embryonic tissue from which the cells originated [120].

## 7. Concluding Remarks

The colocalisation of Hh responsive cells within the adventitial regions of normal vessels, in conjunction with the recapitulation of Hh and Notch components within the medial layer concomitant with the appearance of stem cell derived SMCs following injury, raises the likelihood that a Hh-Notch responsive resident stem cell population is responsible for the generation of neointimal SMCs to support vascular remodelling and neointimal formation during disease progression. Interestingly, previous GWAS studies have reported 13 loci with risk allele frequencies associated with an increase in the risk of cardiovascular disease, one of which is associated with Hh signalling [122]. Notably, only three of the new loci showed significant association with traditional risk factors and the majority lie in gene regions not previously implicated in the pathogenesis of the disease. The fact that local inhibition of a Hh-Notch axis significantly attenuates the generation of neointimal SMCs in animal models of vascular injury only goes to further reinforce the importance of such an axis in controlling vascular cell fate following injury. As more information is forthcoming about the fate of these adventitial and medial progenitor stem cells from vasculogenic zones within the vessel wall, the more likely that the consequences of depletion of these stem cell niches on the structure and function of arteries following injury and during disease progression will be more apparent. The identification of critical soluble factors (such as Hedgehog) and the relevant cell-cell interactions that dictate the behaviour and fate of resident vascular stem cell niches should lead to the development of diagnostic markers and new therapeutic targets for intervention in degenerative/regenerative disease of the arterial wall.

## Figures and Tables

**Table 1:** Expression profiles and locations of resident stem and progenitor cells within the blood vessel wall.

Cell type	Expression profile	Location within the blood vessel wall
AdvSca1^+^ progenitor cells	Sca1^+^, c-kit^+^, Lin^−^, *α*-SMA^−^, SM22^−^, Calponin^−^, and SM-MHC^−^	Tunica adventitia [67]
	Sca1^+^, c-kit^−^, CD140b^+^, CD45^−^, CD68^−^, Shh^+^, Ptch1^+^, Ptch2^+^, Smo^+^, Gli1^+^, Gli2^+^, Gli3^+^, Hhip^+^, Cdo^+^, and Boc^+^	Tunica adventitia [6]

MSCs	CD29^+^, CD44^+^, CD14^−^, and CD45^−^	Tunica media [68]
	CD146^+^, CD34^−^, CD45^−^, CD56^−^, vWF^−^, Pax7^−^, CD144^−^, NG2^+^, *α*-SMA^+^, PDGF-R*β* ^+^, Alkaline Phosphatase^+^, Myogenin^−^, N-Cadherin^−^, Myf5^−^, CD10^+^, CD13^+^, CD44^+^, CD73^+^, CD90^+^, CD105^+^, CD108^+^, CD109^+^, CD140b^+^,CD164^+^, CD166^+^, CD318^+^, CD340^+^, CD349^+^, SSEA-4^+^, HLA-CL^+^, CD106^−^, CD133^−^, CD324^−^, CD326^−^, CD344^−^, HLA-DR^−^	Tunica media [69]
	CD13^+^, CD29^+^, CD44^+^, CD49a^+^, CD49b^+^, CD59^+^, CD73^+^, CD90^+^, CD105^+^, CD31^−^, CD34^+^, CD133^−^, c-kit^−^, CD146^−^, CD45^−^, Desmin^+^, Vimentin^+^, Oct3^−^, Oct4^−^, and NANOG^−^ (additional markers were present in some of the population: NG2^+^ (62% ± 6%), PDGF-R*β* ^+^ (48% ± 2%), Sox2^+^ (75% ± 17%), *α*-SMA^+^ (12% ± 10%), *β*3-tubulin^+^ (58% ± 15%), and Nestin^+^ (20% ± 9%))	Tunica adventitia [70]
	CD13^+^, CD29^+^, CD44^+^, CD54^+^, CD90^+^, HLA Class1^+^, CD14^−^, CD31^−^, CD33^−^, CD34^−^, CD45^−^, CD106^−^, CD133^−^, KDAR^−^, Cadherin-5^−^, and HLA-DR^−^	Tunica media and tunica adventitia [71]

MVSCs	Sox10^+^, Sox1^+^, Snail^+^, Vimentin^+^, Nestin^+^, Sox17^+^, NFM^+^, Peripherin^+^, Brn3a^+^, Phox2b^+^, S100*β* ^+^, CD29^+^, CD44^+^, ki67^+^, *α*-SMA^+^ (*weak*), SM-MHC^−^, CNN1^−^, CD146^−^, Sca1^−^, CD31^−^, CD144^−^, CD34^−^, CD133^−^, c-Kit^−^, Flk-1^−^, and Sox2^−^	Tunica media and tunica adventitia [19]

**Table 2:** Hh-ligand responsive Sca1^+^ progenitor cells and MSCs derived from multiple tissues.

Cell type	Tissue-derived	Hh-ligand responsive
Sca1^+^ progenitor cells	Mouse bronchioalveolar-derived [81]	+
	Mouse BM-derived [15, 82]	+

MSCs	Human adipose-derived [83]	+
	Human umbilical cord blood-derived [84]	+
	Human endometrium-derived [85]	+
	Human BM-derived [86]	+
